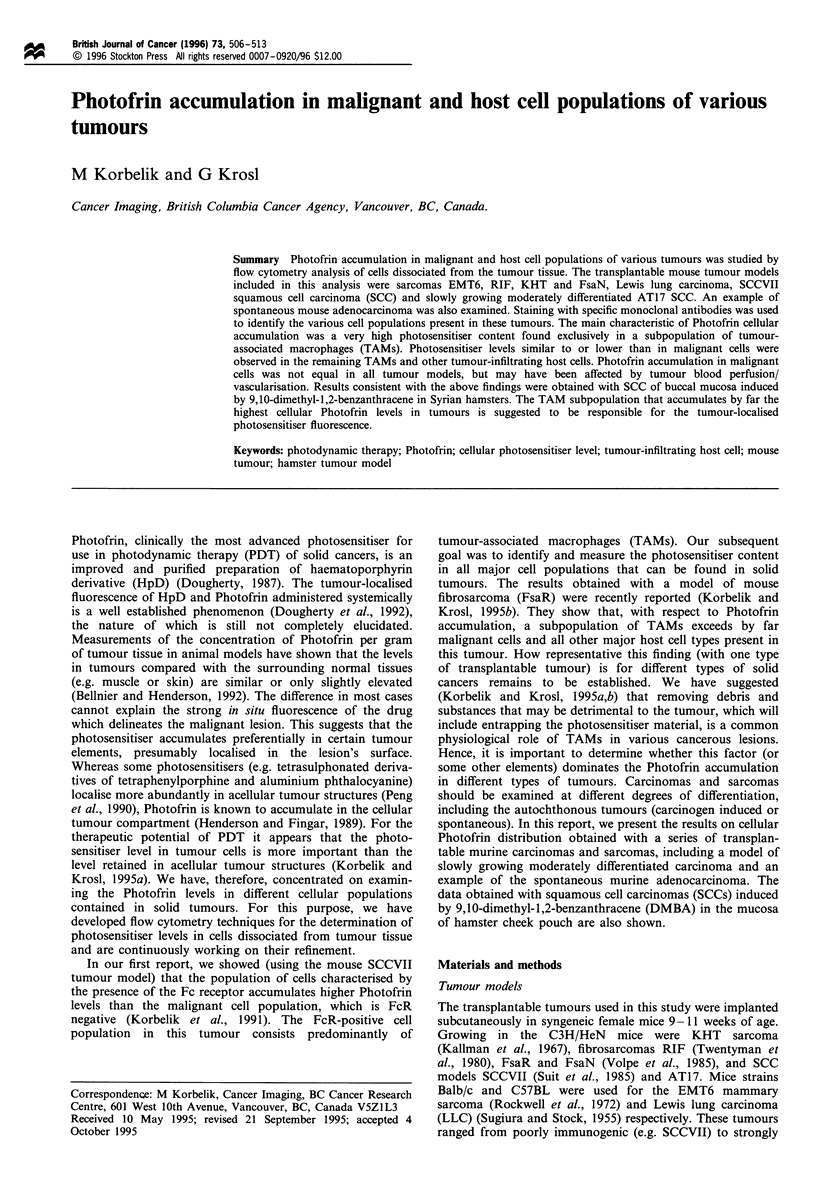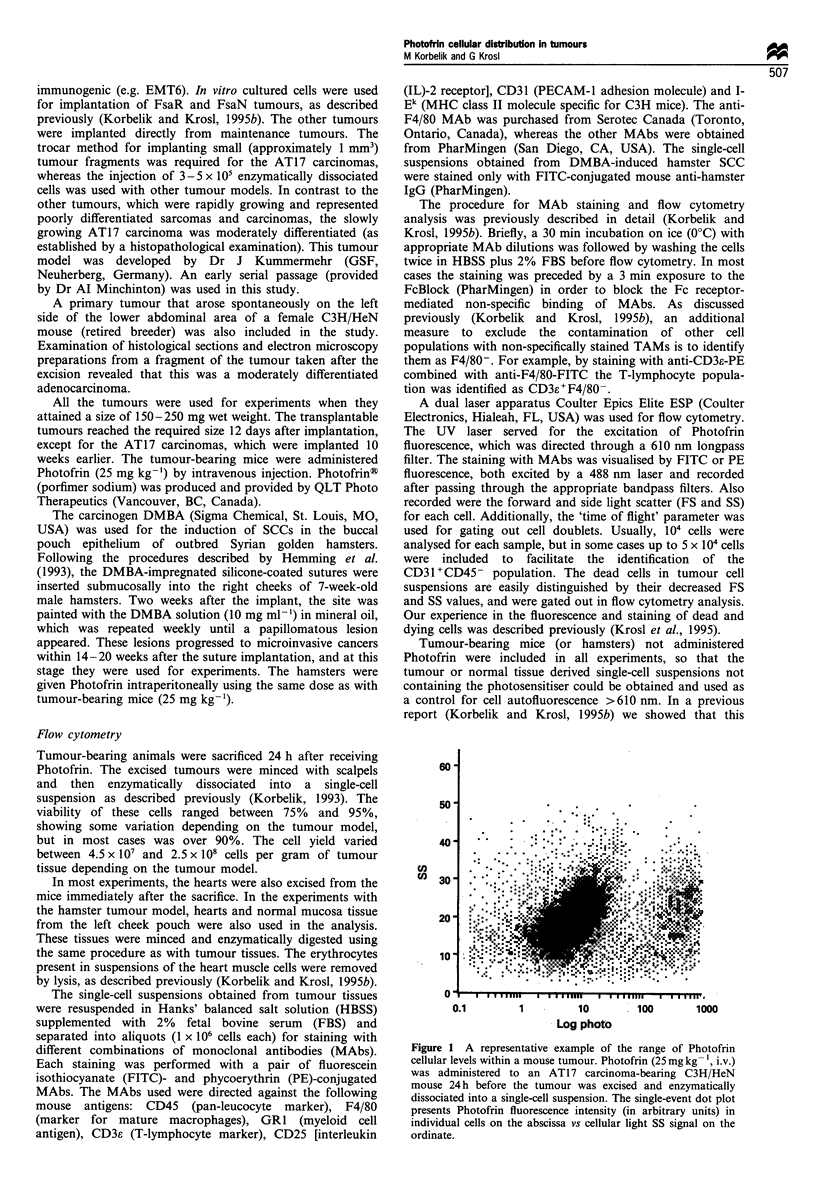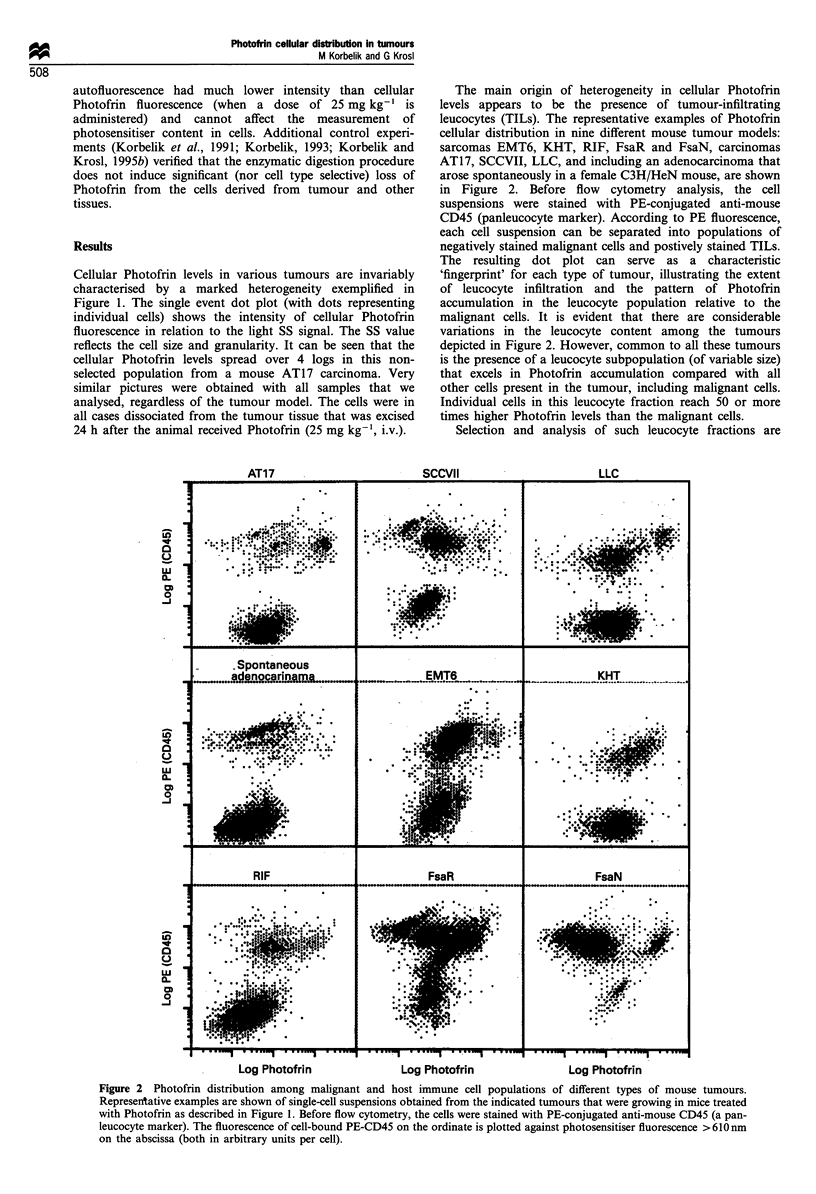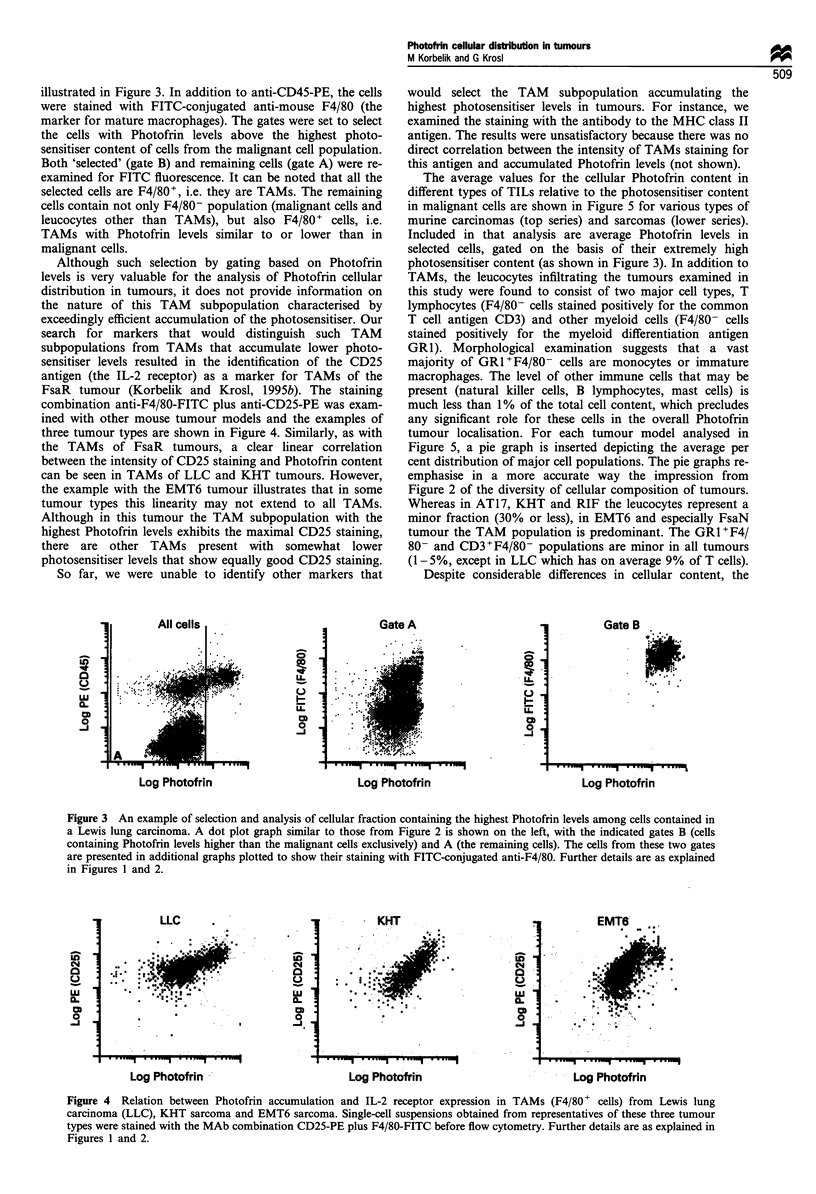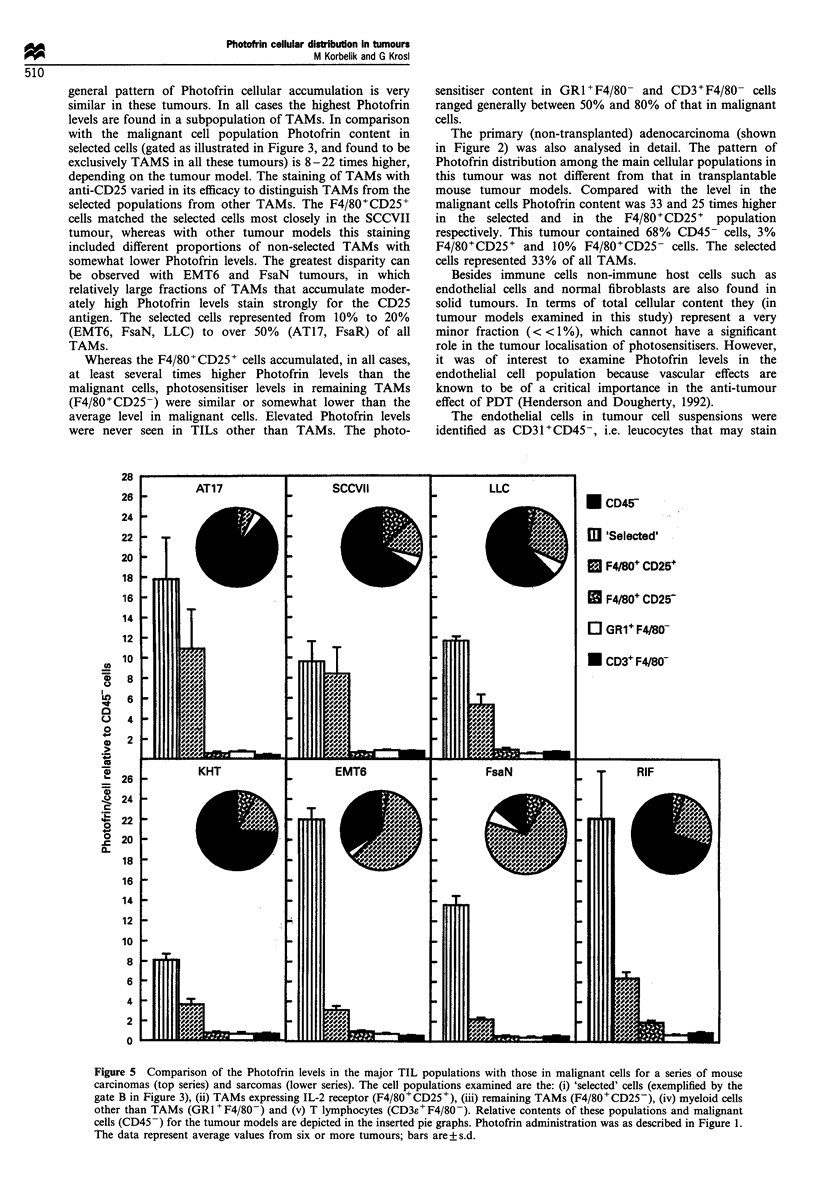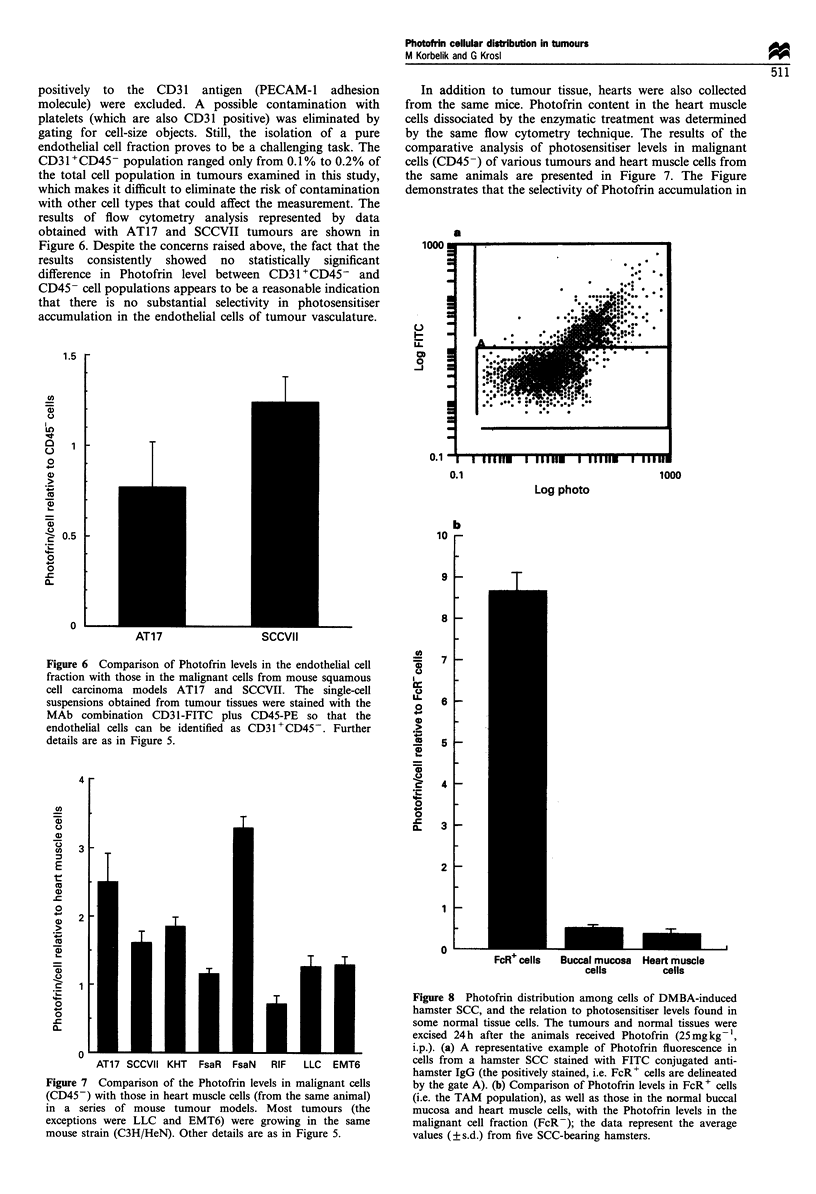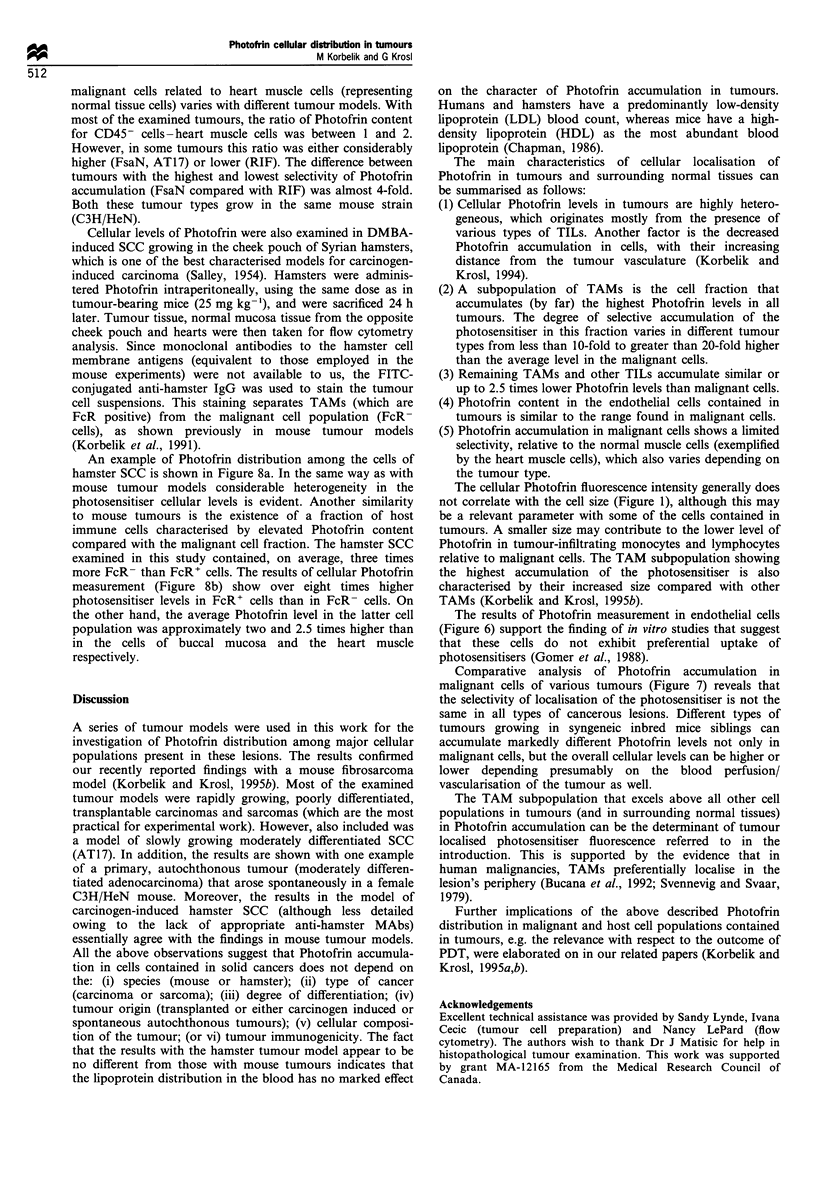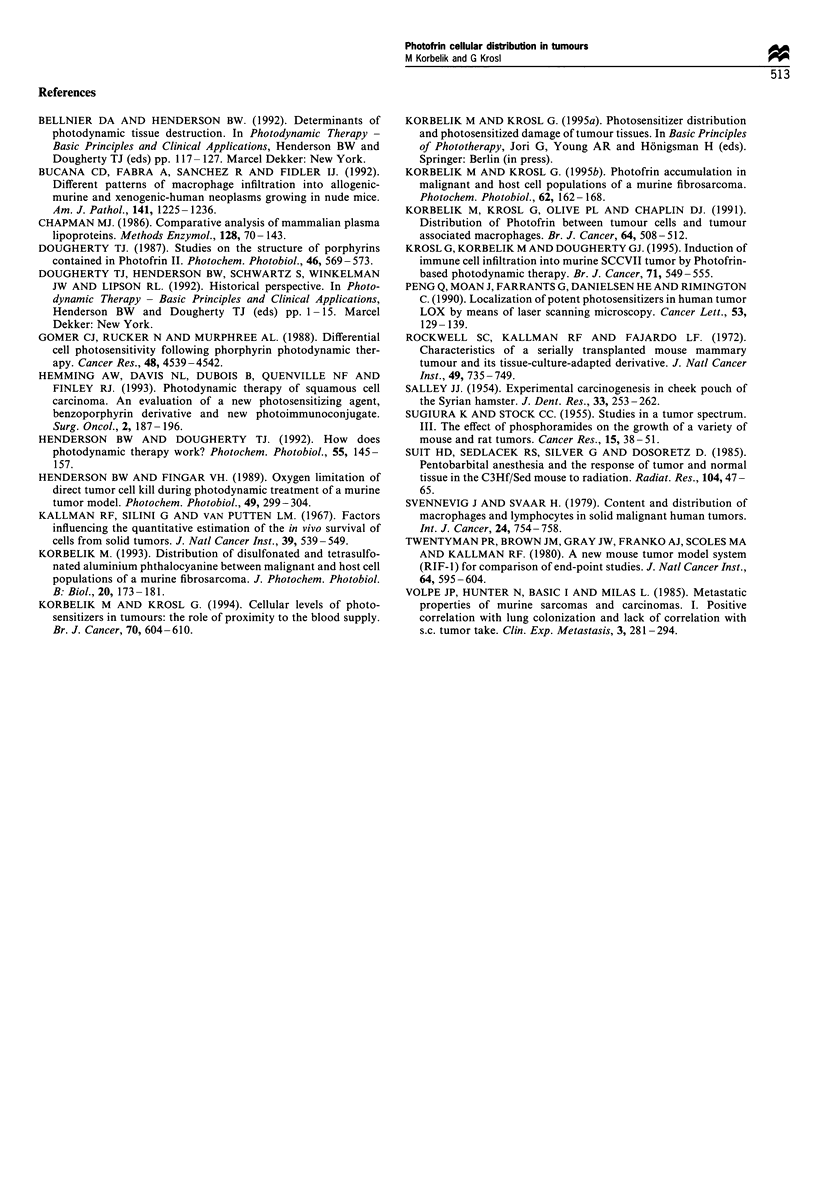# Photofrin accumulation in malignant and host cell populations of various tumours.

**DOI:** 10.1038/bjc.1996.88

**Published:** 1996-02

**Authors:** M. Korbelik, G. Krosl

**Affiliations:** Cancer Imaging, British Columbia Cancer Agency, Vancouver, BC, Canada.

## Abstract

Photofrin accumulation in malignant and host cell populations of various tumours was studied by flow cytometry analysis of cells dissociated from the tumour tissue. The transplantable mouse tumour models included in this analysis were sarcomas EMT6, RIF, KHT and FsaN, Lewis lung carcinoma, SCCVII squamous cell carcinoma (SCC) and slowly growing moderately differentiated AT17 SCC. An example of spontaneous mouse adenocarcinoma was also examined. Staining with specific monoclonal antibodies was used to identify the various cell populations present in these tumours. The main characteristic of Photofrin cellular accumulation was a very high photosensitiser content found exclusively in a subpopulation of tumour-associated macrophages (TAMs). Photosensitiser levels similar to or lower than in malignant cells were observed in the remaining TAMs and other tumour-infiltrating host cells. Photofrin accumulation in malignant cells was not equal in all tumour models, but may have been affected by tumour blood perfusion/vascularisation. Results consistent with the above findings were obtained with SCC of buccal mucosa induced by 9,10-dimethyl-1,2-benzanthracene in Syrian hamsters. The TAM subpopulation that accumulates by far the highest cellular Photofrin levels in tumours is suggested to be responsible for the tumour-localised photosensitiser fluorescence.


British Journal of Cancer (1996) 73, 506-513

© 1996 Stockton Press All rights reserved 0007-0920/96 $12.00

### Photofrin accumulation in malignant and host cell populations of various tumours

M Korbelik and G Krosl

*Cancer Imaging, British Columbia Cancer Agency, Vancouver, BC, Canada.*

**Summary** Photofrin accumulation in malignant and host cell populations of various tumours was studied by flow cytometry analysis of cells dissociated from the tumour tissue. The transplantable mouse tumour models included in this analysis were sarcomas EMT6, RIF, KHT and FsaN, Lewis lung carcinoma, SCCVII squamous cell carcinoma (SCC) and slowly growing moderately differentiated AT17 SCC. An example of spontaneous mouse adenocarcinoma was also examined. Staining with specific monoclonal antibodies was used to identify the various cell populations present in these tumours. The main characteristic of Photofrin cellular accumulation was a very high photosensitiser content found exclusively in a subpopulation of tumour-associated macrophages (TAMs). Photosensitiser levels similar to or lower than in malignant cells were observed in the remaining TAMs and other tumour-infiltrating host cells. Photofrin accumulation in malignant cells was not equal in all tumour models, but may have been affected by tumour blood perfusion/vascularisation. Results consistent with the above findings were obtained with SCC of buccal mucosa induced by 9,10-dimethyl-1,2-benzanthracene in Syrian hamsters. The TAM subpopulation that accumulates by far the highest cellular Photofrin levels in tumours is suggested to be responsible for the tumour-localised photosensitiser fluorescence.

**Keywords:** photodynamic therapy; Photofrin; cellular photosensitiser level; tumour-infiltrating host cell; mouse tumour; hamster tumour model

Photofrin, clinically the most advanced photosensitiser for use in photodynamic therapy (PDT) of solid cancers, is an improved and purified preparation of haematoporphyrin derivative (HpD) (Dougherty, 1987). The tumour-localised fluorescence of HpD and Photofrin administered systemically is a well established phenomenon (Dougherty *et al.*, 1992), the nature of which is still not completely elucidated. Measurements of the concentration of Photofrin per gram of tumour tissue in animal models have shown that the levels in tumours compared with the surrounding normal tissues (e.g. muscle or skin) are similar or only slightly elevated (Bellnier and Henderson, 1992). The difference in most cases cannot explain the strong *in situ* fluorescence of the drug which delineates the malignant lesion. This suggests that the photosensitiser accumulates preferentially in certain tumour elements, presumably localised in the lesion's surface. Whereas some photosensitisers (e.g. tetrasulphonated derivatives of tetraphenylporphine and aluminium phthalocyanine) localise more abundantly in acellular tumour structures (Peng *et al.*, 1990), Photofrin is known to accumulate in the cellular tumour compartment (Henderson and Fingar, 1989). For the therapeutic potential of PDT it appears that the photosensitiser level in tumour cells is more important than the level retained in acellular tumour structures (Korbelik and Krosl, 1995*a*). We have, therefore, concentrated on examining the Photofrin levels in different cellular populations contained in solid tumours. For this purpose, we have developed flow cytometry techniques for the determination of photosensitiser levels in cells dissociated from tumour tissue and are continuously working on their refinement.

In our first report, we showed (using the mouse SCCVII tumour model) that the population of cells characterised by the presence of the Fc receptor accumulates higher Photofrin levels than the malignant cell population, which is FcR negative (Korbelik *et al.*, 1991). The FcR-positive cell population in this tumour consists predominantly of tumour-associated macrophages (TAMs). Our subsequent goal was to identify and measure the photosensitiser content in all major cell populations that can be found in solid tumours. The results obtained with a model of mouse fibrosarcoma (FsaR) were recently reported (Korbelik and Krosl, 1995*b*). They show that, with respect to Photofrin accumulation, a subpopulation of TAMs exceeds by far malignant cells and all other major host cell types present in this tumour. How representative this finding (with one type of transplantable tumour) is for different types of solid cancers remains to be established. We have suggested (Korbelik and Krosl, 1995*a*,*b*) that removing debris and substances that may be detrimental to the tumour, which will include entrapping the photosensitiser material, is a common physiological role of TAMs in various cancerous lesions. Hence, it is important to determine whether this factor (or some other elements) dominates the Photofrin accumulation in different types of tumours. Carcinomas and sarcomas should be examined at different degrees of differentiation, including the autochthonous tumours (carcinogen induced or spontaneous). In this report, we present the results on cellular Photofrin distribution obtained with a series of transplantable murine carcinomas and sarcomas, including a model of slowly growing moderately differentiated carcinoma and an example of the spontaneous murine adenocarcinoma. The data obtained with squamous cell carcinomas (SCCs) induced by 9,10-dimethyl-1,2-benzanthracene (DMBA) in the mucosa of hamster cheek pouch are also shown.

### Materials and methods

### *Tumour models*

The transplantable tumours used in this study were implanted subcutaneously in syngeneic female mice 9-11 weeks of age. Growing in the C3H/HeN mice were KHT sarcoma (Kallman *et al.*, 1967), fibrosarcomas RIF (Twentyman *et al.*, 1980), FsaR and FsaN (Volpe *et al.*, 1985), and SCC models SCCVII (Suit *et al.*, 1985) and AT17. Mice strains Balb/c and C57BL were used for the EMT6 mammary sarcoma (Rockwell *et al.*, 1972) and Lewis lung carcinoma (LLC) (Sugiura and Stock, 1955) respectively. These tumours ranged from poorly immunogenic (e.g. SCCVII) to strongly

Correspondence: M Korbelik, Cancer Imaging, BC Cancer Research Centre, 601 West 10th Avenue, Vancouver, BC, Canada V5Z1L3
Received 10 May 1995; revised 21 September 1995; accepted 4 October 1995

immunogenic (e.g. EMT6). *In vitro* cultured cells were used for implantation of FsaR and FsaN tumours, as described previously (Korbelik and Krosl, 1995*b*). The other tumours were implanted directly from maintenance tumours. The trocar method for implanting small (approximately 1 $mm^3$) tumour fragments was required for the AT17 carcinomas, whereas the injection of $3-5\times10^5$ enzymatically dissociated cells was used with other tumour models. In contrast to the other tumours, which were rapidly growing and represented poorly differentiated sarcomas and carcinomas, the slowly growing AT17 carcinoma was moderately differentiated (as established by a histopathological examination). This tumour model was developed by Dr J Kummermehr (GSF, Neuherberg, Germany). An early serial passage (provided by Dr AI Minchinton) was used in this study.

A primary tumour that arose spontaneously on the left side of the lower abdominal area of a female C3H/HeN mouse (retired breeder) was also included in the study. Examination of histological sections and electron microscopy preparations from a fragment of the tumour taken after the excision revealed that this was a moderately differentiated adenocarcinoma.

All the tumours were used for experiments when they attained a size of 150–250 mg wet weight. The transplantable tumours reached the required size 12 days after implantation, except for the AT17 carcinomas, which were implanted 10 weeks earlier. The tumour-bearing mice were administered Photofrin (25 mg $kg^{-1}$) by intravenous injection. Photofrin® (porfimer sodium) was produced and provided by QLT Photo Therapeutics (Vancouver, BC, Canada).

The carcinogen DMBA (Sigma Chemical, St. Louis, MO, USA) was used for the induction of SCCs in the buccal pouch epithelium of outbred Syrian golden hamsters. Following the procedures described by Hemming *et al.* (1993), the DMBA-impregnated silicone-coated sutures were inserted submucosally into the right cheeks of 7-week-old male hamsters. Two weeks after the implant, the site was painted with the DMBA solution (10 mg $ml^{-1}$) in mineral oil, which was repeated weekly until a papillomatous lesion appeared. These lesions progressed to microinvasive cancers within 14–20 weeks after the suture implantation, and at this stage they were used for experiments. The hamsters were given Photofrin intraperitoneally using the same dose as with tumour-bearing mice (25 mg $kg^{-1}$).

### *Flow cytometry*

Tumour-bearing animals were sacrificed 24 h after receiving Photofrin. The excised tumours were minced with scalpels and then enzymatically dissociated into a single-cell suspension as described previously (Korbelik, 1993). The viability of these cells ranged between 75% and 95%, showing some variation depending on the tumour model, but in most cases was over 90%. The cell yield varied between $4.5\times10^7$ and $2.5\times10^8$ cells per gram of tumour tissue depending on the tumour model.

In most experiments, the hearts were also excised from the mice immediately after the sacrifice. In the experiments with the hamster tumour model, hearts and normal mucosa tissue from the left cheek pouch were also used in the analysis. These tissues were minced and enzymatically digested using the same procedure as with tumour tissues. The erythrocytes present in suspensions of the heart muscle cells were removed by lysis, as described previously (Korbelik and Krosl, 1995*b*).

The single-cell suspensions obtained from tumour tissues were resuspended in Hanks' balanced salt solution (HBSS) supplemented with 2% fetal bovine serum (FBS) and separated into aliquots ($1\times10^6$ cells each) for staining with different combinations of monoclonal antibodies (MAbs). Each staining was performed with a pair of fluorescein isothiocyanate (FITC)- and phycoerythrin (PE)-conjugated MAbs. The MAbs used were directed against the following mouse antigens: CD45 (pan-leucocyte marker), F4/80 (marker for mature macrophages), GR1 (myeloid cell antigen), CD3ε (T-lymphocyte marker), CD25 [interleukin (IL)-2 receptor], CD31 (PECAM-1 adhesion molecule) and I-$E^k$ (MHC class II molecule specific for C3H mice). The anti-F4/80 MAb was purchased from Serotec Canada (Toronto, Ontario, Canada), whereas the other MAbs were obtained from PharMingen (San Diego, CA, USA). The single-cell suspensions obtained from DMBA-induced hamster SCC were stained only with FITC-conjugated mouse anti-hamster IgG (PharMingen).

The procedure for MAb staining and flow cytometry analysis was previously described in detail (Korbelik and Krosl, 1995*b*). Briefly, a 30 min incubation on ice (0°C) with appropriate MAb dilutions was followed by washing the cells twice in HBSS plus 2% FBS before flow cytometry. In most cases the staining was preceded by a 3 min exposure to the FcBlock (PharMingen) in order to block the Fc receptor-mediated non-specific binding of MAbs. As discussed previously (Korbelik and Krosl, 1995*b*), an additional measure to exclude the contamination of other cell populations with non-specifically stained TAMs is to identify them as $F4/80^-$. For example, by staining with anti-CD3ε-PE combined with anti-F4/80-FITC the T-lymphocyte population was identified as $CD3\varepsilon^+F4/80^-$.

A dual laser apparatus Coulter Epics Elite ESP (Coulter Electronics, Hialeah, FL, USA) was used for flow cytometry. The UV laser served for the excitation of Photofrin fluorescence, which was directed through a 610 nm longpass filter. The staining with MAbs was visualised by FITC or PE fluorescence, both excited by a 488 nm laser and recorded after passing through the appropriate bandpass filters. Also recorded were the forward and side light scatter (FS and SS) for each cell. Additionally, the 'time of flight' parameter was used for gating out cell doublets. Usually, $10^4$ cells were analysed for each sample, but in some cases up to $5\times10^4$ cells were included to facilitate the identification of the $CD31^+CD45^-$ population. The dead cells in tumour cell suspensions are easily distinguished by their decreased FS and SS values, and were gated out in flow cytometry analysis. Our experience in the fluorescence and staining of dead and dying cells was described previously (Krosl *et al.*, 1995).

Tumour-bearing mice (or hamsters) not administered Photofrin were included in all experiments, so that the tumour or normal tissue derived single-cell suspensions not containing the photosensitiser could be obtained and used as a control for cell autofluorescence >610 nm. In a previous report (Korbelik and Krosl, 1995*b*) we showed that this

**Figure 1** A representative example of the range of Photofrin cellular levels within a mouse tumour. Photofrin (25 mg $kg^{-1}$, i.v.) was administered to an AT17 carcinoma-bearing C3H/HeN mouse 24 h before the tumour was excised and enzymatically dissociated into a single-cell suspension. The single-event dot plot presents Photofrin fluorescence intensity (in arbitrary units) in individual cells on the abscissa *vs* cellular light SS signal on the ordinate.

autofluorescence had much lower intensity than cellular Photofrin fluorescence (when a dose of 25 mg $kg^{-1}$ is administered) and cannot affect the measurement of photosensitiser content in cells. Additional control experiments (Korbelik *et al.*, 1991; Korbelik, 1993; Korbelik and Krosl, 1995*b*) verified that the enzymatic digestion procedure does not induce significant (nor cell type selective) loss of Photofrin from the cells derived from tumour and other tissues.

### Results

Cellular Photofrin levels in various tumours are invariably characterised by a marked heterogeneity exemplified in Figure 1. The single event dot plot (with dots representing individual cells) shows the intensity of cellular Photofrin fluorescence in relation to the light SS signal. The SS value reflects the cell size and granularity. It can be seen that the cellular Photofrin levels spread over 4 logs in this non-selected population from a mouse AT17 carcinoma. Very similar pictures were obtained with all samples that we analysed, regardless of the tumour model. The cells were in all cases dissociated from the tumour tissue that was excised 24 h after the animal received Photofrin (25 mg $kg^{-1}$, i.v.).

The main origin of heterogeneity in cellular Photofrin levels appears to be the presence of tumour-infiltrating leucocytes (TILs). The representative examples of Photofrin cellular distribution in nine different mouse tumour models: sarcomas EMT6, KHT, RIF, FsaR and FsaN, carcinomas AT17, SCCVII, LLC, and including an adenocarcinoma that arose spontaneously in a female C3H/HeN mouse, are shown in Figure 2. Before flow cytometry analysis, the cell suspensions were stained with PE-conjugated anti-mouse CD45 (panleucocyte marker). According to PE fluorescence, each cell suspension can be separated into populations of negatively stained malignant cells and postively stained TILs. The resulting dot plot can serve as a characteristic 'fingerprint' for each type of tumour, illustrating the extent of leucocyte infiltration and the pattern of Photofrin accumulation in the leucocyte population relative to the malignant cells. It is evident that there are considerable variations in the leucocyte content among the tumours depicted in Figure 2. However, common to all these tumours is the presence of a leucocyte subpopulation (of variable size) that excels in Photofrin accumulation compared with all other cells present in the tumour, including malignant cells. Individual cells in this leucocyte fraction reach 50 or more times higher Photofrin levels than the malignant cells.

Selection and analysis of such leucocyte fractions are

**Figure 2** Photofrin distribution among malignant and host immune cell populations of different types of mouse tumours. Representative examples are shown of single-cell suspensions obtained from the indicated tumours that were growing in mice treated with Photofrin as described in Figure 1. Before flow cytometry, the cells were stained with PE-conjugated anti-mouse CD45 (a pan-leucocyte marker). The fluorescence of cell-bound PE-CD45 on the ordinate is plotted against photosensitiser fluorescence >610 nm on the abscissa (both in arbitrary units per cell).

illustrated in Figure 3. In addition to anti-CD45-PE, the cells were stained with FITC-conjugated anti-mouse F4/80 (the marker for mature macrophages). The gates were set to select the cells with Photofrin levels above the highest photosensitiser content of cells from the malignant cell population. Both 'selected' (gate B) and remaining cells (gate A) were re-examined for FITC fluorescence. It can be noted that all the selected cells are F4/80$^+$, i.e. they are TAMs. The remaining cells contain not only F4/80$^-$ population (malignant cells and leucocytes other than TAMs), but also F4/80$^+$ cells, i.e. TAMs with Photofrin levels similar to or lower than in malignant cells.

Although such selection by gating based on Photofrin levels is very valuable for the analysis of Photofrin cellular distribution in tumours, it does not provide information on the nature of this TAM subpopulation characterised by exceedingly efficient accumulation of the photosensitiser. Our search for markers that would distinguish such TAM subpopulations from TAMs that accumulate lower photosensitiser levels resulted in the identification of the CD25 antigen (the IL-2 receptor) as a marker for TAMs of the FsaR tumour (Korbelik and Krosl, 1995*b*). The staining combination anti-F4/80-FITC plus anti-CD25-PE was examined with other mouse tumour models and the examples of three tumour types are shown in Figure 4. Similarly, as with the TAMs of FsaR tumours, a clear linear correlation between the intensity of CD25 staining and Photofrin content can be seen in TAMs of LLC and KHT tumours. However, the example with the EMT6 tumour illustrates that in some tumour types this linearity may not extend to all TAMs. Although in this tumour the TAM subpopulation with the highest Photofrin levels exhibits the maximal CD25 staining, there are other TAMs present with somewhat lower photosensitiser levels that show equally good CD25 staining.

So far, we were unable to identify other markers that would select the TAM subpopulation accumulating the highest photosensitiser levels in tumours. For instance, we examined the staining with the antibody to the MHC class II antigen. The results were unsatisfactory because there was no direct correlation between the intensity of TAMs staining for this antigen and accumulated Photofrin levels (not shown).

The average values for the cellular Photofrin content in different types of TILs relative to the photosensitiser content in malignant cells are shown in Figure 5 for various types of murine carcinomas (top series) and sarcomas (lower series). Included in that analysis are average Photofrin levels in selected cells, gated on the basis of their extremely high photosensitiser content (as shown in Figure 3). In addition to TAMs, the leucocytes infiltrating the tumours examined in this study were found to consist of two major cell types, T lymphocytes (F4/80$^-$ cells stained positively for the common T cell antigen CD3) and other myeloid cells (F4/80$^-$ cells stained positively for the myeloid differentiation antigen GR1). Morphological examination suggests that a vast majority of GR1$^+$F4/80$^-$ cells are monocytes or immature macrophages. The level of other immune cells that may be present (natural killer cells, B lymphocytes, mast cells) is much less than 1% of the total cell content, which precludes any significant role for these cells in the overall Photofrin tumour localisation. For each tumour model analysed in Figure 5, a pie graph is inserted depicting the average per cent distribution of major cell populations. The pie graphs re-emphasise in a more accurate way the impression from Figure 2 of the diversity of cellular composition of tumours. Whereas in AT17, KHT and RIF the leucocytes represent a minor fraction (30% or less), in EMT6 and especially FsaN tumour the TAM population is predominant. The GR1$^+$F4/80$^-$ and CD3$^+$F4/80$^-$ populations are minor in all tumours (1–5%, except in LLC which has on average 9% of T cells).

Despite considerable differences in cellular content, the

**Figure 3** An example of selection and analysis of cellular fraction containing the highest Photofrin levels among cells contained in a Lewis lung carcinoma. A dot plot graph similar to those from Figure 2 is shown on the left, with the indicated gates B (cells containing Photofrin levels higher than the malignant cells exclusively) and A (the remaining cells). The cells from these two gates are presented in additional graphs plotted to show their staining with FITC-conjugated anti-F4/80. Further details are as explained in Figures 1 and 2.

**Figure 4** Relation between Photofrin accumulation and IL-2 receptor expression in TAMs (F4/80$^+$ cells) from Lewis lung carcinoma (LLC), KHT sarcoma and EMT6 sarcoma. Single-cell suspensions obtained from representatives of these three tumour types were stained with the MAb combination CD25-PE plus F4/80-FITC before flow cytometry. Further details are as explained in Figures 1 and 2.

general pattern of Photofrin cellular accumulation is very similar in these tumours. In all cases the highest Photofrin levels are found in a subpopulation of TAMs. In comparison with the malignant cell population Photofrin content in selected cells (gated as illustrated in Figure 3, and found to be exclusively TAMS in all these tumours) is 8–22 times higher, depending on the tumour model. The staining of TAMs with anti-CD25 varied in its efficacy to distinguish TAMs from the selected populations from other TAMs. The F4/80$^+$CD25$^+$ cells matched the selected cells most closely in the SCCVII tumour, whereas with other tumour models this staining included different proportions of non-selected TAMs with somewhat lower Photofrin levels. The greatest disparity can be observed with EMT6 and FsaN tumours, in which relatively large fractions of TAMs that accumulate moderately high Photofrin levels stain strongly for the CD25 antigen. The selected cells represented from 10% to 20% (EMT6, FsaN, LLC) to over 50% (AT17, FsaR) of all TAMs.

Whereas the F4/80$^+$CD25$^+$ cells accumulated, in all cases, at least several times higher Photofrin levels than the malignant cells, photosensitiser levels in remaining TAMs (F4/80$^+$CD25$^-$) were similar or somewhat lower than the average level in malignant cells. Elevated Photofrin levels were never seen in TILs other than TAMs. The photosensitiser content in GR1$^+$F4/80$^-$ and CD3$^+$F4/80$^-$ cells ranged generally between 50% and 80% of that in malignant cells.

The primary (non-transplanted) adenocarcinoma (shown in Figure 2) was also analysed in detail. The pattern of Photofrin distribution among the main cellular populations in this tumour was not different from that in transplantable mouse tumour models. Compared with the level in the malignant cells Photofrin content was 33 and 25 times higher in the selected and in the F4/80$^+$CD25$^+$ population respectively. This tumour contained 68% CD45$^-$ cells, 3% F4/80$^+$CD25$^+$ and 10% F4/80$^+$CD25$^-$ cells. The selected cells represented 33% of all TAMs.

Besides immune cells non-immune host cells such as endothelial cells and normal fibroblasts are also found in solid tumours. In terms of total cellular content they (in tumour models examined in this study) represent a very minor fraction (<<1%), which cannot have a significant role in the tumour localisation of photosensitisers. However, it was of interest to examine Photofrin levels in the endothelial cell population because vascular effects are known to be of a critical importance in the anti-tumour effect of PDT (Henderson and Dougherty, 1992).

The endothelial cells in tumour cell suspensions were identified as CD31$^+$CD45$^-$, i.e. leucocytes that may stain

**Figure 5** Comparison of the Photofrin levels in the major TIL populations with those in malignant cells for a series of mouse carcinomas (top series) and sarcomas (lower series). The cell populations examined are the: (i) 'selected' cells (exemplified by the gate B in Figure 3), (ii) TAMs expressing IL-2 receptor (F4/80$^+$CD25$^+$), (iii) remaining TAMs (F4/80$^+$CD25$^-$), (iv) myeloid cells other than TAMs (GR1$^+$F4/80$^-$) and (v) T lymphocytes (CD3ε$^+$F4/80$^-$). Relative contents of these populations and malignant cells (CD45$^-$) for the tumour models are depicted in the inserted pie graphs. Photofrin administration was as described in Figure 1. The data represent average values from six or more tumours; bars are ± s.d.

positively to the CD31 antigen (PECAM-1 adhesion molecule) were excluded. A possible contamination with platelets (which are also CD31 positive) was eliminated by gating for cell-size objects. Still, the isolation of a pure endothelial cell fraction proves to be a challenging task. The $CD31^+CD45^-$ population ranged only from 0.1% to 0.2% of the total cell population in tumours examined in this study, which makes it difficult to eliminate the risk of contamination with other cell types that could affect the measurement. The results of flow cytometry analysis represented by data obtained with AT17 and SCCVII tumours are shown in Figure 6. Despite the concerns raised above, the fact that the results consistently showed no statistically significant difference in Photofrin level between $CD31^+CD45^-$ and $CD45^-$ cell populations appears to be a reasonable indication that there is no substantial selectivity in photosensitiser accumulation in the endothelial cells of tumour vasculature.

**Figure 6** Comparison of Photofrin levels in the endothelial cell fraction with those in the malignant cells from mouse squamous cell carcinoma models AT17 and SCCVII. The single-cell suspensions obtained from tumour tissues were stained with the MAb combination CD31-FITC plus CD45-PE so that the endothelial cells can be identified as $CD31^+CD45^-$. Further details are as in Figure 5.

**Figure 7** Comparison of the Photofrin levels in malignant cells ($CD45^-$) with those in heart muscle cells (from the same animal) in a series of mouse tumour models. Most tumours (the exceptions were LLC and EMT6) were growing in the same mouse strain (C3H/HeN). Other details are as in Figure 5.

In addition to tumour tissue, hearts were also collected from the same mice. Photofrin content in the heart muscle cells dissociated by the enzymatic treatment was determined by the same flow cytometry technique. The results of the comparative analysis of photosensitiser levels in malignant cells ($CD45^-$) of various tumours and heart muscle cells from the same animals are presented in Figure 7. The Figure demonstrates that the selectivity of Photofrin accumulation in

**Figure 8** Photofrin distribution among cells of DMBA-induced hamster SCC, and the relation to photosensitiser levels found in some normal tissue cells. The tumours and normal tissues were excised 24 h after the animals received Photofrin ($25\,mg\,kg^{-1}$, i.p.). (**a**) A representative example of Photofrin fluorescence in cells from a hamster SCC stained with FITC conjugated anti-hamster IgG (the positively stained, i.e. $FcR^+$ cells are delineated by the gate A). (**b**) Comparison of Photofrin levels in $FcR^+$ cells (i.e. the TAM population), as well as those in the normal buccal mucosa and heart muscle cells, with the Photofrin levels in the malignant cell fraction ($FcR^-$); the data represent the average values ($\pm$s.d.) from five SCC-bearing hamsters.

malignant cells related to heart muscle cells (representing normal tissue cells) varies with different tumour models. With most of the examined tumours, the ratio of Photofrin content for $CD45^-$ cells–heart muscle cells was between 1 and 2. However, in some tumours this ratio was either considerably higher (FsaN, AT17) or lower (RIF). The difference between tumours with the highest and lowest selectivity of Photofrin accumulation (FsaN compared with RIF) was almost 4-fold. Both these tumour types grow in the same mouse strain (C3H/HeN).

Cellular levels of Photofrin were also examined in DMBA-induced SCC growing in the cheek pouch of Syrian hamsters, which is one of the best characterised models for carcinogen-induced carcinoma (Salley, 1954). Hamsters were administered Photofrin intraperitoneally, using the same dose as in tumour-bearing mice (25 mg $kg^{-1}$), and were sacrificed 24 h later. Tumour tissue, normal mucosa tissue from the opposite cheek pouch and hearts were then taken for flow cytometry analysis. Since monoclonal antibodies to the hamster cell membrane antigens (equivalent to those employed in the mouse experiments) were not available to us, the FITC-conjugated anti-hamster IgG was used to stain the tumour cell suspensions. This staining separates TAMs (which are FcR positive) from the malignant cell population ($FcR^-$ cells), as shown previously in mouse tumour models (Korbelik *et al.*, 1991).

An example of Photofrin distribution among the cells of hamster SCC is shown in Figure 8a. In the same way as with mouse tumour models considerable heterogeneity in the photosensitiser cellular levels is evident. Another similarity to mouse tumours is the existence of a fraction of host immune cells characterised by elevated Photofrin content compared with the malignant cell fraction. The hamster SCC examined in this study contained, on average, three times more $FcR^-$ than $FcR^+$ cells. The results of cellular Photofrin measurement (Figure 8b) show over eight times higher photosensitiser levels in $FcR^+$ cells than in $FcR^-$ cells. On the other hand, the average Photofrin level in the latter cell population was approximately two and 2.5 times higher than in the cells of buccal mucosa and the heart muscle respectively.

### Discussion

A series of tumour models were used in this work for the investigation of Photofrin distribution among major cellular populations present in these lesions. The results confirmed our recently reported findings with a mouse fibrosarcoma model (Korbelik and Krosl, 1995*b*). Most of the examined tumour models were rapidly growing, poorly differentiated, transplantable carcinomas and sarcomas (which are the most practical for experimental work). However, also included was a model of slowly growing moderately differentiated SCC (AT17). In addition, the results are shown with one example of a primary, autochthonous tumour (moderately differentiated adenocarcinoma) that arose spontaneously in a female C3H/HeN mouse. Moreover, the results in the model of carcinogen-induced hamster SCC (although less detailed owing to the lack of appropriate anti-hamster MAbs) essentially agree with the findings in mouse tumour models. All the above observations suggest that Photofrin accumulation in cells contained in solid cancers does not depend on the: (i) species (mouse or hamster); (ii) type of cancer (carcinoma or sarcoma); (iii) degree of differentiation; (iv) tumour origin (transplanted or either carcinogen induced or spontaneous autochthonous tumours); (v) cellular composition of the tumour; (or vi) tumour immunogenicity. The fact that the results with the hamster tumour model appear to be no different from those with mouse tumours indicates that the lipoprotein distribution in the blood has no marked effect on the character of Photofrin accumulation in tumours. Humans and hamsters have a predominantly low-density lipoprotein (LDL) blood count, whereas mice have a high-density lipoprotein (HDL) as the most abundant blood lipoprotein (Chapman, 1986).

The main characteristics of cellular localisation of Photofrin in tumours and surrounding normal tissues can be summarised as follows:

(1) Cellular Photofrin levels in tumours are highly heterogeneous, which originates mostly from the presence of various types of TILs. Another factor is the decreased Photofrin accumulation in cells, with their increasing distance from the tumour vasculature (Korbelik and Krosl, 1994).
(2) A subpopulation of TAMs is the cell fraction that accumulates (by far) the highest Photofrin levels in all tumours. The degree of selective accumulation of the photosensitiser in this fraction varies in different tumour types from less than 10-fold to greater than 20-fold higher than the average level in the malignant cells.
(3) Remaining TAMs and other TILs accumulate similar or up to 2.5 times lower Photofrin levels than malignant cells.
(4) Photofrin content in the endothelial cells contained in tumours is similar to the range found in malignant cells.
(5) Photofrin accumulation in malignant cells shows a limited selectivity, relative to the normal muscle cells (exemplified by the heart muscle cells), which also varies depending on the tumour type.

The cellular Photofrin fluorescence intensity generally does not correlate with the cell size (Figure 1), although this may be a relevant parameter with some of the cells contained in tumours. A smaller size may contribute to the lower level of Photofrin in tumour-infiltrating monocytes and lymphocytes relative to malignant cells. The TAM subpopulation showing the highest accumulation of the photosensitiser is also characterised by their increased size compared with other TAMs (Korbelik and Krosl, 1995*b*).

The results of Photofrin measurement in endothelial cells (Figure 6) support the finding of *in vitro* studies that suggest that these cells do not exhibit preferential uptake of photosensitisers (Gomer *et al.*, 1988).

Comparative analysis of Photofrin accumulation in malignant cells of various tumours (Figure 7) reveals that the selectivity of localisation of the photosensitiser is not the same in all types of cancerous lesions. Different types of tumours growing in syngeneic inbred mice siblings can accumulate markedly different Photofrin levels not only in malignant cells, but the overall cellular levels can be higher or lower depending presumably on the blood perfusion/vascularisation of the tumour as well.

The TAM subpopulation that excels above all other cell populations in tumours (and in surrounding normal tissues) in Photofrin accumulation can be the determinant of tumour localised photosensitiser fluorescence referred to in the introduction. This is supported by the evidence that in human malignancies, TAMs preferentially localise in the lesion's periphery (Bucana *et al.*, 1992; Svennevig and Svaar, 1979).

Further implications of the above described Photofrin distribution in malignant and host cell populations contained in tumours, e.g. the relevance with respect to the outcome of PDT, were elaborated on in our related papers (Korbelik and Krosl, 1995*a*,*b*).

**Acknowledgements**
Excellent technical assistance was provided by Sandy Lynde, Ivana Cecic (tumour cell preparation) and Nancy LePard (flow cytometry). The authors wish to thank Dr J Matisic for help in histopathological tumour examination. This work was supported by grant MA-12165 from the Medical Research Council of Canada.

### References

BELLNIER DA AND HENDERSON BW. (1992). Determinants of photodynamic tissue destruction. In *Photodynamic Therapy – Basic Principles and Clinical Applications*, Henderson BW and Dougherty TJ (eds) pp. 117–127. Marcel Dekker: New York.

BUCANA CD, FABRA A, SANCHEZ R AND FIDLER IJ. (1992). Different patterns of macrophage infiltration into allogenic-murine and xenogenic-human neoplasms growing in nude mice. *Am. J. Pathol.*, **141,** 1225–1236.

CHAPMAN MJ. (1986). Comparative analysis of mammalian plasma lipoproteins. *Methods Enzymol.*, **128,** 70–143.

DOUGHERTY TJ. (1987). Studies on the structure of porphyrins contained in Photofrin II. *Photochem. Photobiol.*, **46,** 569–573.

DOUGHERTY TJ, HENDERSON BW, SCHWARTZ S, WINKELMAN JW AND LIPSON RL. (1992). Historical perspective. In *Photodynamic Therapy – Basic Principles and Clinical Applications*, Henderson BW and Dougherty TJ (eds) pp. 1–15. Marcel Dekker: New York.

GOMER CJ, RUCKER N AND MURPHREE AL. (1988). Differential cell photosensitivity following phorphyrin photodynamic therapy. *Cancer Res.*, **48,** 4539–4542.

HEMMING AW, DAVIS NL, DUBOIS B, QUENVILLE NF AND FINLEY RJ. (1993). Photodynamic therapy of squamous cell carcinoma. An evaluation of a new photosensitizing agent, benzoporphyrin derivative and new photoimmunoconjugate. *Surg. Oncol.*, **2,** 187–196.

HENDERSON BW AND DOUGHERTY TJ. (1992). How does photodynamic therapy work? *Photochem. Photobiol.*, **55,** 145–157.

HENDERSON BW AND FINGAR VH. (1989). Oxygen limitation of direct tumor cell kill during photodynamic treatment of a murine tumor model. *Photochem. Photobiol.*, **49,** 299–304.

KALLMAN RF, SILINI G AND VAN PUTTEN LM. (1967). Factors influencing the quantitative estimation of the *in vivo* survival of cells from solid tumors. *J. Natl Cancer Inst.*, **39,** 539–549.

KORBELIK M. (1993). Distribution of disulfonated and tetrasulfonated aluminium phthalocyanine between malignant and host cell populations of a murine fibrosarcoma. *J. Photochem. Photobiol. B: Biol.*, **20,** 173–181.

KORBELIK M AND KROSL G. (1994). Cellular levels of photosensitizers in tumours: the role of proximity to the blood supply. *Br. J. Cancer*, **70,** 604–610.

KORBELIK M AND KROSL G. (1995*a*). Photosensitizer distribution and photosensitized damage of tumour tissues. In *Basic Principles of Phototherapy*, Jori G, Young AR and Hönigsman H (eds). Springer: Berlin (in press).

KORBELIK M AND KROSL G. (1995*b*). Photofrin accumulation in malignant and host cell populations of a murine fibrosarcoma. *Photochem. Photobiol.*, **62,** 162–168.

KORBELIK M, KROSL G, OLIVE PL AND CHAPLIN DJ. (1991). Distribution of Photofrin between tumour cells and tumour associated macrophages. *Br. J. Cancer*, **64,** 508–512.

KROSL G, KORBELIK M AND DOUGHERTY GJ. (1995). Induction of immune cell infiltration into murine SCCVII tumor by Photofrin-based photodynamic therapy. *Br. J. Cancer*, **71,** 549–555.

PENG Q, MOAN J, FARRANTS G, DANIELSEN HE AND RIMINGTON C. (1990). Localization of potent photosensitizers in human tumor LOX by means of laser scanning microscopy. *Cancer Lett.*, **53,** 129–139.

ROCKWELL SC, KALLMAN RF AND FAJARDO LF. (1972). Characteristics of a serially transplanted mouse mammary tumour and its tissue-culture-adapted derivative. *J. Natl Cancer Inst.*, **49,** 735–749.

SALLEY JJ. (1954). Experimental carcinogenesis in cheek pouch of the Syrian hamster. *J. Dent. Res.*, **33,** 253–262.

SUGIURA K AND STOCK CC. (1955). Studies in a tumor spectrum. III. The effect of phosphoramides on the growth of a variety of mouse and rat tumors. *Cancer Res.*, **15,** 38–51.

SUIT HD, SEDLACEK RS, SILVER G AND DOSORETZ D. (1985). Pentobarbital anesthesia and the response of tumor and normal tissue in the C3Hf/Sed mouse to radiation. *Radiat. Res.*, **104,** 47–65.

SVENNEVIG J AND SVAAR H. (1979). Content and distribution of macrophages and lymphocytes in solid malignant human tumors. *Int. J. Cancer*, **24,** 754–758.

TWENTYMAN PR, BROWN JM, GRAY JW, FRANKO AJ, SCOLES MA AND KALLMAN RF. (1980). A new mouse tumor model system (RIF-1) for comparison of end-point studies. *J. Natl Cancer Inst.*, **64,** 595–604.

VOLPE JP, HUNTER N, BASIC I AND MILAS L. (1985). Metastatic properties of murine sarcomas and carcinomas. I. Positive correlation with lung colonization and lack of correlation with s.c. tumor take. *Clin. Exp. Metastasis*, **3,** 281–294.